# A Regional Approach to a Global Challenge: A Case Report of Caudal Anesthesia for Anoplasty in Tetralogy of Fallot With Pulmonary Atresia

**DOI:** 10.1002/ccr3.71735

**Published:** 2025-12-28

**Authors:** Prince Barnawal, Rukesh Yadav, Sandeep Khatri, K. C. Jyoti, Sudarshan Acharya

**Affiliations:** ^1^ Maharajgunj Medical Campus, Institute of Medicine Kathmandu Nepal; ^2^ Department of Anaesthesiology Tribhuvan University Teaching Hospital Kathmandu Nepal

**Keywords:** case report, caudal anesthesia, neonate, pulmonary atresia, tetralogy of Fallot

## Abstract

Patients with Tetralogy of Fallot (TOF) with pulmonary atresia present high perioperative risks during non‐cardiac surgeries, making anesthetic management challenging. Caudal anesthesia combined with sedation offers a safe and effective alternative when planned carefully, supported by a sound understanding of the patient's unique physiology and meticulous perioperative monitoring.

## Graphical abstract

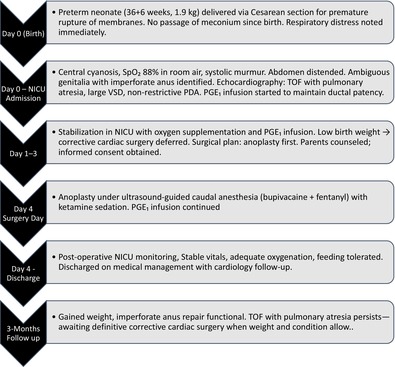

## 1 Introduction

Tetralogy of Fallot (TOF) is the most common cyanotic congenital heart disease. Approximately 15% of the patients with pulmonary atresia, the most severe form, rely on patent ductus arteriosus (PDA) for survival [1]. Prostaglandin infusion keeps the PDA open until corrective surgery. Mortality rate is 3%–6% in these neonates [2].

The presence of cyanotic heart disease (CHD) significantly increases the mortality and perioperative risk in such patients undergoing non‐cardiac surgical intervention [3]. A review revealed that the mortality risk in patients with CHD undergoing non‐cardiac surgery was double as compared with a similar population without CHD [4].

When dealing with patients with CHD who need non‐cardiac surgery, anaesthesiologists face a variety of challenges due to the complex cardiac lesion, the patient's compensatory potential, the urgency of the procedure, and other coexisting conditions [5].

Despite the risk, anesthesia can still be given to a newborn with a complex cardiac disease when they present with a general surgical emergency with careful precaution. The type of anesthesia chosen depends on the specific patient factors [6]. Caudal anesthesia offers a safe alternative for non‐cardiac surgery [3]. In this report, we managed a case of a neonate with TOF, pulmonary atresia, and PDA who underwent anoplasty under caudal anesthesia successfully.

## 2 Patient Presentation

**FIGURE 1:**
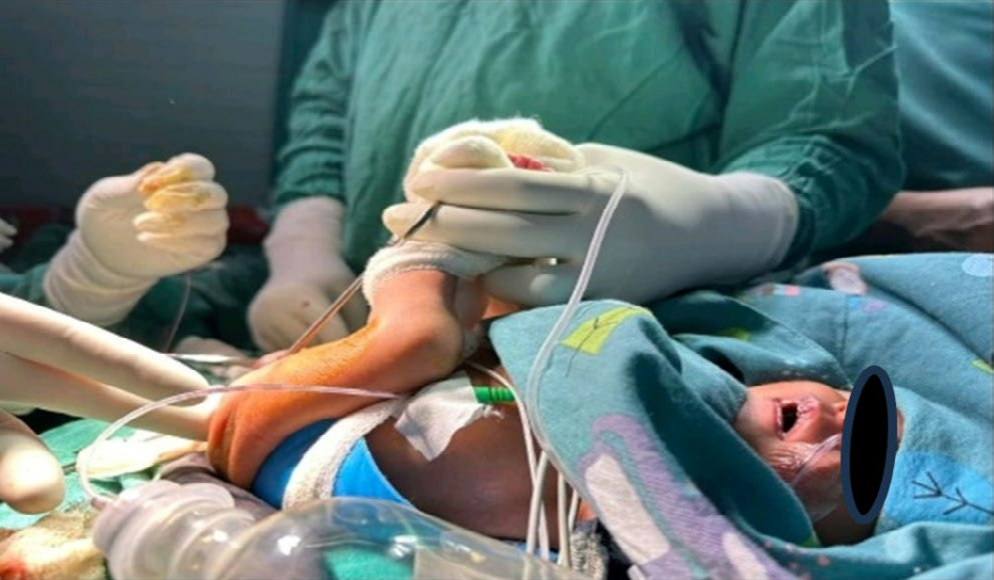
Patient in a supine lithotomy position undergoing anoplasty procedure under caudal anesthesia with ketamine sedation with oxygen supplementation via nasal cannula. The monitoring setup includes ECG, SpO2, and non‐invasive blood pressure. The surgical site is prepped and draped, and the sterile field is maintained by the operating team wearing gloves and gowns.

**FIGURE 2:**
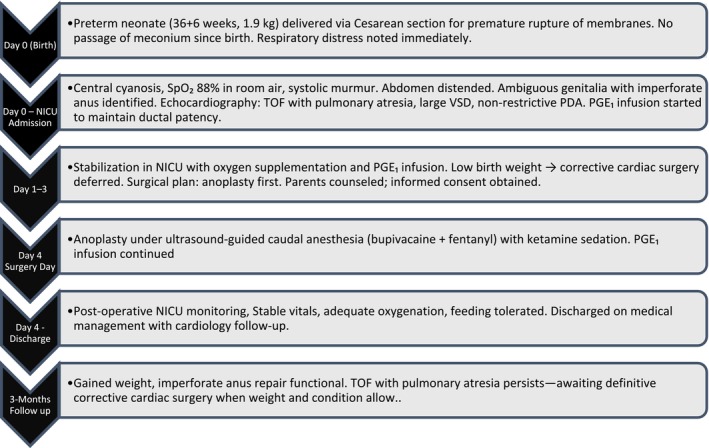
Timeline of the patient's clinical course from birth to follow‐up: [birth, diagnosis, NICU admission, prostaglandin infusion, surgery date, post‐op NICU stay, discharge, and 3‐month follow‐up].

A 4‐day‐old, 1.9 kg, preterm neonate (36 + 6 weeks' gestation) born via Cesarean delivery for Premature rupture of the membrane, presented with the chief complaint of not passing stool since birth. She also had symptoms of difficulty breathing since birth. The baby was admitted in the neonatal intensive care unit due to respiratory distress. The general physical examination revealed central cyanosis, a pulse rate of 129 beats per minute (bpm), blood pressure (BP) of 80/40 mm of mercury (mmHg), and oxygen saturation (Sp02) of 88% in the room air. On auscultation, the baby was found to have a systolic ejection murmur at all auscultatory areas. The abdomen was distended with a girth of 27 cm. On local examination, ambiguous genitalia with imperforate anus were observed. Echocardiography revealed TOF with pulmonary atresia, a non‐restrictive 3 mm PDA with bidirectional flow and a systolic gradient of 20 mmHg, and a large subaortic ventricular septal defect (VSD) with bidirectional shunting. To maintain the patency of the PDA, prostaglandin E1 (PGE1) infusion was started in the NICU. Ultrasonography (USG) of the abdomen and the pelvis confirmed low anal atresia. Definitive corrective surgery for TOF was deferred due to low BW as per cardiology consultation, so it was more reasonable to correct the imperforate anus first. Detailed informed consent was obtained from the parents regarding the potential risks of the planned surgery and the anesthesia in the setting of cyanotic heart disease of the patient. The baby was planned for anoplasty under caudal anesthesia, and the surgery was performed under caudal anesthesia as shown in Figure 1. A detailed timeline of the patient's clinical course from birth to follow‐up is depicted in Figure 2.

## 3 Anesthetic Management

Patient was positioned in the left lateral position for caudal block administration. Using ultrasound guidance, the caudal epidural space was accessed with a 26‐G cannula. 2 mL of 0.25% bupivacaine with 2 mcg of fentanyl was administered as the anesthetic agent. IV Ketamine 1 mg/kg was given for sedation. Oxygen was insufflated throughout the procedure, and PGE1 infusion 0.07 mcg/kg/min was continued throughout the perioperative period. Towards the end of the procedure, SpO2 suddenly dropped to 44%, BP dropped to 50/25 mmHg, heart rate decreased to 88 bpm. Bag and mask ventilation was done, 8 mL of balanced salt solution was given as bolus and phenylephrine 2.5 mcg was administered as a bolus twice. Vitals were stabilized and the child was then shifted to the NICU. The surgery lasted for 25 min.

## 4 Discussion

TOF with pulmonary atresia are prone to chronic hypoxemia, hypercarbia, respiratory alkalosis, cyanosis, polycythaemia, hyperventilation, arrhythmias, heart failure, thrombosis, infections, and cyanotic spells [6, 7]. These complications contribute to the increased mortality in such patients undergoing non‐cardiac surgery [6].

We did not consider general anesthesia in the first place in our patients due to several reasons. General anesthesia can exacerbate right‐to‐left shunting due to systemic vasodilation, reduce SVR, and involve airway manipulation, all of which may provoke cyanotic spells [4]. Moreover, we avoided the need for airway manipulation in the neonate by choosing caudal anesthesia. At times, CHD is linked to other anomalies such as VACTERL association, which often make airway difficult [8].

Single‐shot caudal anesthesia with ketamine sedation with oxygen supplementation was done with careful monitoring. Use of caudal anesthesia avoided the need for airway manipulation and the use of polypharmacy that may alter the balance of SVR. We have to be ready to manage cyanotic spells during surgery, which may be caused by various insults. The main goals to manage TOF with pulmonary atresia with PDA include maintaining SVR and decreasing pulmonary vascular resistance (PVR) by avoiding hypoxia, hypercapnia, hypothermia, maintaining volume, and contractility [2, 9]. The reduction of systemic vascular resistance (SVR) caused by caudal anesthesia is somewhat countered by an increase in SVR caused by ketamine, thus aligning with the hemodynamic goal in this case. We used ketamine for sedation as it maintains the SVR. Ketamine was chosen over other sedatives such as dexmedetomidine or midazolam because of its well‐documented ability to increase systemic vascular resistance (SVR) while maintaining myocardial contractility, thus supporting systemic perfusion in cyanotic heart lesions [10].

CHD patients undergoing non‐cardiac surgery are among the high‐risk groups often necessitating high dependency care [5, 9]. Attention to every single factor that may potentially lead to cyanotic spells should be taken care of. Even adequate pain management reduces the chance of cyanotic spells by reducing the sympathetic stimulation [11]. In fact, the goals of anesthesia in the patients with CHD should focus on maintaining or increasing the SVR and reducing the PVR [12]. The factors affecting these two parameters should be dealt with in order to avoid the cyanotic spells and its dreadful complications like arrhythmias [6, 12].

It is crucial to avoid metabolic abnormalities, tachycardia, and dehydration during the intraoperative and postoperative phases in TOF patients undergoing surgery. These variables frequently cause cyanotic episodes as a result of hypertrophied pulmonary infundibulum spasms. In these situations, arterial blood gas (ABG) analysis is useful since end‐tidal carbon dioxide values underestimate PaCO_2_ and pulse oximetry overestimates arterial oxygen saturation as saturation decreases [13]. It is well recognized that transesophageal echocardiography (TEE) is highly helpful for assessing intracardiac shunting and can potentially help avoid cyanotic spells [14].

The spells are recognized by hypotension, ischemic changes on ECG, and sharply declining SpO2. The spells are often avoided and managed by considering the mentioned entities [9]. The treatment of cyanotic spells on the table includes increasing inspired oxygen, administering a fluid bolus, using opiates to deepen anesthesia, and an alpha agonist to increase SVR and decrease right‐to‐left shunt [12, 13]. In our case, the likely precipitating factor was sympathetic blockade resulting from caudal anesthesia. The child was receiving maintenance fluid preoperatively, and the calculated amount of fluid was given intraoperatively. There was minimal third‐space loss due to the nature of the surgery, so hypovolemia was less likely the cause. Early recognition and intervention, including oxygen supplementation, positive pressure ventilation, fluid resuscitation, and phenylephrine, restored haemodynamic stability. We opted for phenylephrine over ephedrine due to its predominant alpha‐adrenergic agonist effect, which increases SVR without significantly increasing heart rate—a desirable effect in cyanotic heart disease where tachycardia can worsen shunting [15].

Other case reports have used a combination of general and regional anesthetic procedures for surgical intervention in patients with CHD. Since there is no set gold standard, a risk–benefit analysis that is specific to the patient's current situation should be performed. Neuraxial anesthesia by causing sympathectomy may not be tolerable in patients with complex CHD lesions and severe pulmonary hypertension [5]. Likewise, there are drawbacks to general anesthesia. Among these are a possible drop in SVR brought on by the vasodilating effect of anesthetic medications and an increase in PVR brought on by hypoxia, hypercarbia, acidosis, and hypothermia [6]. Reduced pulmonary flow and subsequent cyanosis is another common result of positive pressure ventilation's impact on the pulmonary vasculature [6].

### 4.1 Patient and Family Perspective

The parents expressed relief that the surgical team could perform the procedure without the need for general anesthesia, which they had been concerned about due to the baby's heart condition. They appreciated the clear communication and continuous updates during the perioperative period and reported satisfaction with the recovery process.

## 5 Conclusion

Regional anesthesia with sedation offers a safe and optimal option for performing non‐cardiac surgery in complex cardiac lesions like TOF with PA with PDA.

### 5.1 Key Takeaways

- Main hemodynamic goal is maintaining or increasing the SVR and reducing the PVR.
- Sympathetic blockade caused by caudal anesthesia is mitigated by increased SVR caused by ketamine aligning with the hemodynamic goal.
- Be prepared to promptly recognize and treat cyanotic spells

Careful planning and a firm grasp of physiological and pharmacological principles are essential for the effective completion of the surgery in patients with CHD.

## Author Contributions

**Prince Barnawal:** conceptualization, formal analysis, visualization, writing – original draft, writing – review and editing. **Rukesh Yadav:** writing – original draft, writing – review and editing. **Sandeep Khatri:** conceptualization, visualization, writing – original draft, writing – review and editing. **K. C. Jyoti:** supervision, visualization, writing – original draft, writing – review and editing. **Sudarshan Acharya:** conceptualization, supervision, visualization, writing – original draft, writing – review and editing.

## Funding

The authors have nothing to report.

## Disclosure

*Guarantor*: Sandeep Khatri.

*Declaration*: All the authors declare that the information provided here is accurate to the best of our knowledge.

## Ethics Statement

Ethical approval was exempted by BP Koirala Institute of Health Science for case reports.

## Consent

Written informed consent was obtained from the patient for publication.

## Conflicts of Interest

The authors declare no conflicts of interest.

## Data Availability

Data sharing is not applicable to this case report as no new data were created or analyzed in this study.
